# Screen-Printed Electrodes for the Voltammetric Sensing of Benzotriazoles in Water

**DOI:** 10.3390/s20071839

**Published:** 2020-03-26

**Authors:** Alessandra Muschietti, Núria Serrano, Cristina Ariño, M. Silvia Díaz-Cruz, José Manuel Díaz-Cruz

**Affiliations:** 1 Department of Chemical Engineering and Analytical Chemistry, University of Barcelona, Martí i Franquès 1-11, 08028 Barcelona, Spain; muschiettialessandra@gmail.com (A.M.); nuria.serrano@ub.edu (N.S.); cristina.arino@ub.edu (C.A.); 2 Institut de Recerca de l’Aigua (IdRA), University of Barcelona, 08028 Barcelona, Spain; 3 Department of Environmental Chemistry, Institute of Environmental Assessment and Water Research—Severo Ochoa Excellence Center (IDAEA), Spanish Council of Scientific Research (CSIC), Jordi Girona 18-26, 08034 Barcelona, Spain; sdcqam@cid.csic.es

**Keywords:** voltammetric sensors, screen-printed electrodes, benzotriazole, tolyltriazole

## Abstract

Benzotriazoles (BZTs) are high production volume industrial chemicals that are used in various applications such as corrosion inhibitors, antifreeze agents, and UV radiation stabilizers. Given their potential ecotoxicological implications for different ecosystems and in human health, as well as their poor biodegradability, they are of increasing concern. In this study, a new voltammetric method using commercial screen-printed electrodes (SPEs) has been developed for the sensing of BZTs in water samples to help in their environmental monitoring. To this end, different types of SPEs based on carbon nanoallotropes and copper were tested under several experimental conditions to determine the two BZTs most frequently detected in the environment: 1H-benzotriazole (BZT) and 5-methyl-1H-benzotriazole (Me-BZT, tolyltriazole) as model compounds for BZTs. Carbon nanofibers electrodes exhibited the best performance, allowing detection limits as low as 0.4 mg L^−1^ for both BZTs, with repeatability and reproducibility of ca. 5%. The applicability of the method was tested through the determination of BZT in spiked drinking water samples, suggesting its suitability for the sensing of samples heavily polluted with BZTs.

## 1. Introduction

Currently, there is a growing interest in anthropogenic pollutants with differing origins and chemical characteristics which are classified as contaminants of emerging concern. They are not yet included in the legislation but potentially may pose an environmental hazard. This is because they are toxic, persistent, tend to bioaccumulate, and are poorly removed during conventional wastewater treatment due to their high solubility, polarity, and limited biodegradability [1,2,3,4,5]. 

Benzotriazoles (BZTs) encompasses a wide group of substances that have been commercialized since the late 1950s. Today they are high production volume industrial chemicals used as UV light stabilizers in many products including food packaging, automotive products, lubricants and grease, dishwasher detergents, anti-icing and defrosting products, textiles, plastics, and rubber materials [6,7,8,9,10,11]. 

BZTs typically appear in the environment as a result of deicing activities and discharges from wastewater treatment plants [5,12,13]. Because of their structural differences and associated wide range of physicochemical properties, they can reach all environmental compartments. Anticorrosive agents such as benzotriazole (BZT) and methyl-triazole (Me-BZT), are highly soluble in water, with values of 20 g L^−1^ and 5 g L^−1^ at 20 °C, respectively, and have been found mainly in natural waters. Due to their high vapor pressure, certain BZTs have also been detected in the air. BZT and Me-BZT were measured in indoor air from houses and public buildings at mean concentrations between 2 and 7 ng m^−3^ [14]. Concentrations in the µg L^−1^ range for BZT and Me-BZT were measured in river water [15,16]. Other studies reported the bioaccumulation of certain BZTs in fish [16,17,18]. BZTs can ultimately accumulate in humans, e.g., in adipose tissues, urine, breast milk, and amniotic fluid [19,20,21]. Acute toxicity due to BZTs was reported in several fish species, zooplankton, and bacteria (ca. 10 mg L^−1^) [22]. Moreover, Cornell et al. reported that toxicity of Me-BZT manifests in at least two ways: through acute toxicity to all tested organisms, and through a decrease in the ability of soil microorganisms to degrade propylene glycol, the primary component of aircraft deicing fluids [23]. Similarly, Cancilla et al. identified BZT and Me-BZT as the two compounds from aircraft deicing fluids which are most toxic to Microtox test microorganisms [24]. Very high concentrations were reported for these compounds in ground and surface waters near airports, for example with values of 126 mg L^−1^ BZT and 198 mg L^−1^ Me-BZT [25]. Quite high concentrations, e.g., 6 mg L^−1^, were also measured in wastewater [26]. These levels are far above the half maximal effective concentration (EC50) estimated values and are sufficiently high to decrease propylene glycol biodegradation rates in the subsurface. Fortunately, the reference concentrations determined in other water ecosystems were below the chronic and acute toxicity values. Nevertheless, their continuous introduction into the environment, together with their low degradability and persistence [12], makes necessary the inclusion of such pollutants into future monitoring and biomonitoring programs. 

The determination of BZTs in the diverse environmental samples is mainly carried out by high-performance liquid chromatography coupled to mass spectrometry (HPLC-MS). This technique provides the high sensitivity and selectivity required for the trace levels at which these compounds occur in the environment [5,6]. However, it has some disadvantages, such as the high cost of instrumentation and operation, relatively large amount of waste generated, need for specialized analysts (due to the complexity of the instrumentation), and the other operations it entails (for example time-consuming pre-treatments).

Electroanalytical methods appear as useful tools for the determination of BZTs considering the advantages of being simple, fast, cost-effective, and suitable for on-site analysis. Regardless, electroanalytical studies on this topic are very scarce. In 1968, Lund et al. [27] explored the polarographic reduction of BZT, and 20 years later Pedersen et al. [28] analyzed the polarographic reduction of some BZTs in protic and aprotic media. Later on, in 2010, the electrochemical behavior of BZT in a glassy carbon electrode was investigated by Lokesh et al. [29] by cyclic voltammetry (CV) at quite high concentrations (> 60 mg L^−1^). In 2016, Zheng et al. [30] studied the voltammetric behavior of BZT by square wave voltammetry in a glassy carbon electrode modified with multiwalled carbon nanotubes and Nafion. More recently (2019), Ababneh et al. [31] investigated the determination of BZT derivatives by differential pulse polarography (DPP) in a static mercury drop electrode. In both cases, voltammetric methods were developed for the determination of BZT with limits of detection (LODs) below the mg L^−1^ level. However, to the best of our knowledge, no more voltammetric methods have been proposed to determine BZTs levels. Moreover, the electrodes used by the authors of [30] and [31] could be problematic. On the one hand, conventional solid electrodes require frequent and time-consuming conditioning and, on the other hand, mercury electrodes are progressively becoming obsolete because of their potential toxicity and the difficult adaptation to flow and on-site conditions.

Under this scenario, the purpose of this work was to develop a voltammetric methodology for BZTs determination using commercial screen-printed electrodes (SPEs). This is a topic that, as far as we know, has not been described yet in the literature. SPEs are disposable and low-cost devices quite reproducible from one unit to the other which are much less toxic than mercury electrodes and which, unlike conventional solid electrodes, do not need tedious polishing and activation procedures prior to measurement. SPEs are obtained by printing on a plastic or ceramic support a series of inks containing the components of the working, reference, and auxiliary electrodes and the required connectors. Screen-printed devices have found promising applications in the environmental analysis because of their suitability for on-site analysis, supported by their linear output, low power requirement, quick response, high sensitivity, and capability to operate at room temperature [32]. Very recently, we have developed and applied successful methodologies for the determination of organic UV filters [33] and pharmaceuticals [34] in water by using SPEs. 

In the present study, we have focused on the applicability of commercial screen-printed electrodes as electrochemical sensors for the detection of BZT and BZT derivatives in water. To this end, screen-printed carbon (SPCE), carbon nanofiber (SPCNFE), multi-walled carbon nanotube (SPCNTE), and copper (SPCuE) electrodes were tested under different experimental conditions to select the best operating conditions for BZT determination in water matrices.

## 2. Materials and Methods

### 2.1. Reagents

All reagents used in the present study were of analytical grade and all solutions were prepared using ultrapure water obtained from an Elix 3 instrument coupled to a Milli-Q system by Millipore (Bedford, MA, USA).

**Figure 1:**
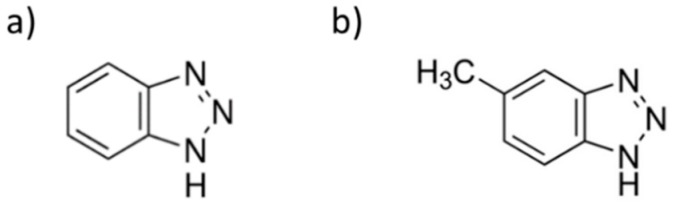
Chemical structure of (**a**) 1H-benzotriazole (BZT) and (**b**) 5-methyl-1H-benzotriazole (Me-BZT).

1-H-benzotriazole of 99% purity (CASn# 95-14-7) was purchased from Sigma-Aldrich (St. Louis, MO, USA), and 5-methyl-1-H-benzotriazole (CASn# 136-85-6, 99%) was purchased from TCI (Zwijndrecht, Belgium) (see Figure 1). Stock solutions of BZT and Me-BZT were prepared by dissolving the solid reagent in purified water and then stored in the fridge at 4 °C and protected from light. Diluted solutions of BZT and Me-BZT were prepared immediately before their use by dissolving a certain amount of stock solution in the electrolyte support.

The buffer solutions used as supporting electrolyte were prepared by adding a diluted solution of sodium hydroxide (Scharlau Chemie S.A., Barcelona) to an appropriate volume of trifluoroacetic acid, purity 99% (Sigma Aldrich, Germany) to adjust pH values (pH = 2.20 when not otherwise indicated) and to achieve a total concentration of trifluoroacetic acid of 0.01 mol L^−1^. 

### 2.2. Instrumentation and Apparatus

Differential pulse voltammetry (DPV) and CV were applied to an electrochemical system consisting of a μAutolab III potentiostat (EcoChemie, The Netherlands) with an IME663 interface attached to the 663 VA Stand system (Metrohm, Switzerland) and controlled by Autolab GPES 4.9 (EcoChemie, The Netherlands) software. The stand was equipped with a screen-printed device purchased from Metrohm-Dropsens (Oviedo, Spain) used as the working electrode, and with an external Ag/AgCl reference electrode by Metrohm (saturated with 3 mol L^−1^ KCl) and an external platinum auxiliary electrode, also from Metrohm, for improved accuracy. All commercially available SPEs tested have a surface of 50.3 mm^2^ and are made of different carbon nanoallotropes: SPCE (reference DRP-110), SPCNTE (reference DRP-110CNT), and SPCNFE (reference DRP-110CNF), as well as a copper nanoallotrope: SPCuE (reference DRP-CU10). As an interface among the SPEs and the potentiostat system, a Metrohm-DropSens DSC connector was used (reference DRP-CAC).

An analytical balance Mettler Toledo (Columbus, USA) AT261 was used to prepare the stock solutions required for the analysis. pH measurements were performed using a Crison (Hach Lange Spain, L’Hospitalet de Llobregat, Spain) Basic-20 pH-meter with a Crison 5011T electrode. Nichiryo Nichipet EX micropipettes (Tokyo, Japan) were used to transfer solutions to the electrochemical cell.

### 2.3. Procedures

For DPV and CV measurements, 25 mL of the buffer solution at the required pH value were placed in a glass voltammetric cell by Metrohm. Calibration plots were obtained by successive additions of BZT or Me-BZT standard solutions into the buffer (blank) solution. For methodology validation purposes, the quantification of BZT in water samples was carried out. Standard addition was applied as the calibration method. When not otherwise indicated, potentials were measured with a differential pulse scan from −0.7 V to −1.4 V, using pulse heights of 50 mV, pulse times of 50 ms, and scan rates of 20 mV s^−1^. All the experiments were performed at room temperature (20 °C).

### 2.4. Data Treatment

Peak heights and areas were measured with GPES version 4.9 software and further calculations were made with EXCEL^®^ program. Sensitivities are given as the slope of the calibration plot. LODs and limits of quantification (LOQs) were computed as 3 and 10 times the standard deviation of the intercept of the calibration line, divided by the slope, respectively. Linearity ranges are given from the LOQ values to the most concentrated standards for which linearity still holds. Repeatability was studied from 10 consecutive measurements at a concentration in the middle of the linearity range and, in the case of SPCNFE electrodes, reproducibility was estimated from the relative standard deviation (RSD) of the slopes of three independent calibration lines.

## 3. Results and Discussion

### 3.1. Preliminary Studies 

**Figure 2:**
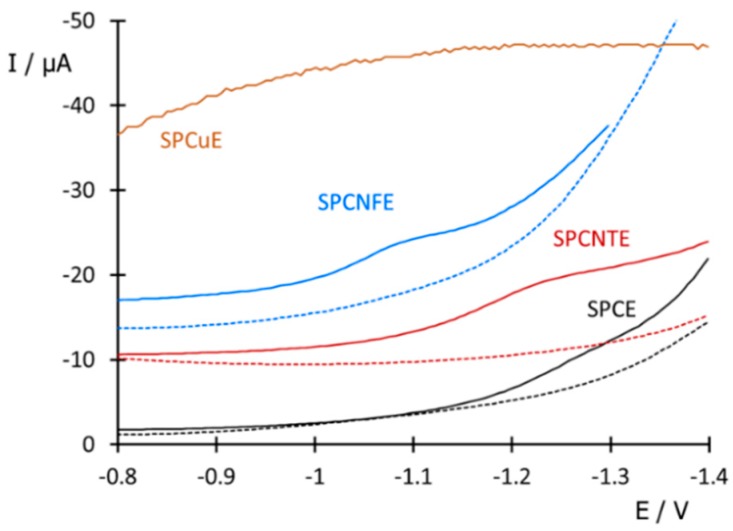
Differential pulse voltammetry (DPV) voltammograms measured for a BZT solution of 5 mg L^−1^ at pH 2.2 with screen-printed electrodes made of carbon (SPCE), carbon nanofibers (SPCNFE), carbon nanotubes (SPCNTE), and copper (SPCuE). Dashed lines show the corresponding measurements for a blank solution.

To obtain the best analytical signal for BZT and Me-BZT determination, different types of SPEs based on both carbon nanoallotropes and copper were tested by DPV. As Figure 2 shows, SPCE provided a poor signal in BZT solutions, hardly different from the voltammogram of the blank, suggesting slow electrode kinetics of the possible faradaic processes generated by BZT. As for SPCuE, it gave a high background current of reduction where no signal due to BZTs could be distinguished, despite the great affinity of copper with BZT described in the literature [35]. In contrast, both SPCNTE and SPCNFE showed a well-defined characteristic reduction peak. As already mentioned in the Introduction section, the use of nanomaterials such as carbon nanotubes or carbon nanofibers improves the sensitivity of the tests because of the special properties they posse, e.g., a high surface area favoring the electrochemical processes taking place on the electrode surface [36]. Therefore, given the preliminary results obtained, further experiments were carried out using SPCNTE and SPCNFE.

**Figure 3:**
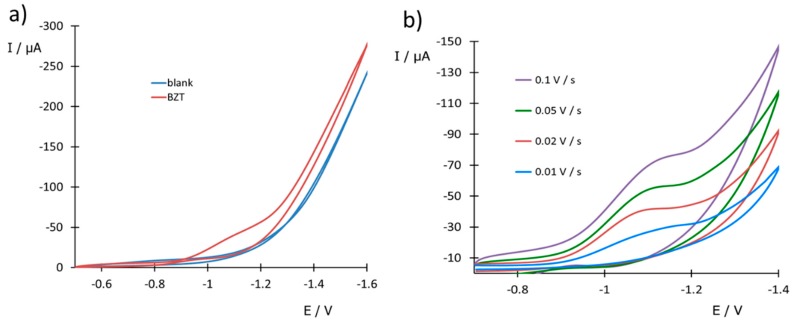
Cyclic voltammetry (CV) voltammograms measured with a SPCNFE in a solution containing 7 mg L^−1^ of BZT at pH 2.2, (**a**) at 0.01 V s^−1^ in comparison with the blank and (**b**) at different scan rates.

A preliminary electrochemical study was carried out by CV. Figure 3a displays typical CV voltammograms obtained with an SPCNFE in the absence and the presence of BZT. In the blank solution, no reduction peak is observed while in the presence of BZT a reduction peak appears centered at −1.1 V. The absence of the corresponding (re)oxidation peak suggests the irreversibility of BZT reduction. In Figure 3b, CV voltammograms of BZT at a concentration of 7 mg L^−1^ and pH 2.2, applying different scan rates from 10 to 100 mV s^−1^, are shown. The above-mentioned absence of anodic peak together with the shift of the cathodic peak towards negative potentials at increasing scan rates confirm the irreversible character of the reduction of BZT. In the case of Me-BZT, very similar CV voltammograms were recorded, with the cathodic signal at almost the same potential (−1.1 V) as that of BZT (figure not shown). This suggests that the presence of the methyl group in the Me-BZT molecule as compared to BZT does not affect the electrochemical process. Moreover, good linearity among reduction peak currents and BZT concentrations was found. Nevertheless, CV is mostly used for diagnosis purposes and has lower sensitivity than DPV, which, as will be discussed later, is more convenient for quantitative analysis. 

**Figure 4:**
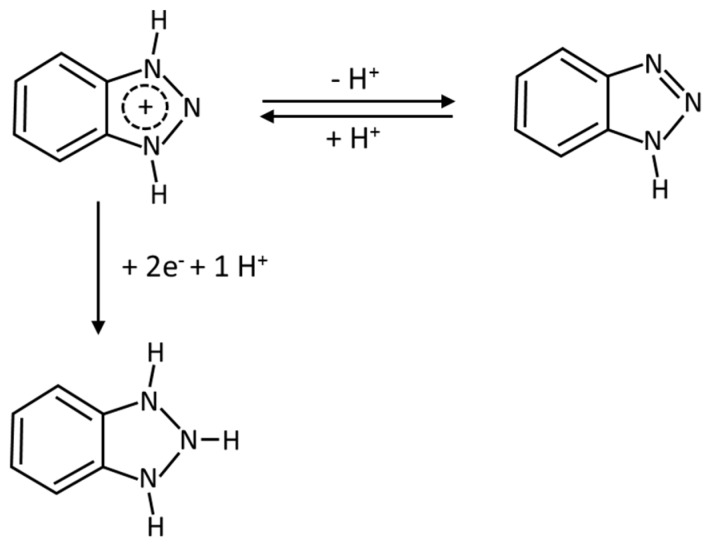
Tautomeric forms and electroreduction process of BZT in acidic media.

As reported in previous studies [29,37], the electrochemical reduction of BZT (pK_a_ 8.2) is a two-electron reaction producing o-aminophenylhydrazine and involving a protonation reaction (Figure 4). Therefore, high concentrations of H^+^-ions (i.e., low pH values) favor the electrochemical reduction of BZT. Nevertheless, acidic pH values also produce an increase of the background current due to the reduction of H^+^-ions at the working electrode. In the present study, a pH value of 2.2 was used as a compromise between high sensitivity and acceptable background current. 

### 3.2. Study by Differential Pulse Voltammetry 

Once CV was applied to confirm the irreversible electrochemical reduction of BZTs at SPCNFE and SPCNTE, DPV was applied to develop and validate a method for the electroanalytical determination of these substances.

**Figure 5:**
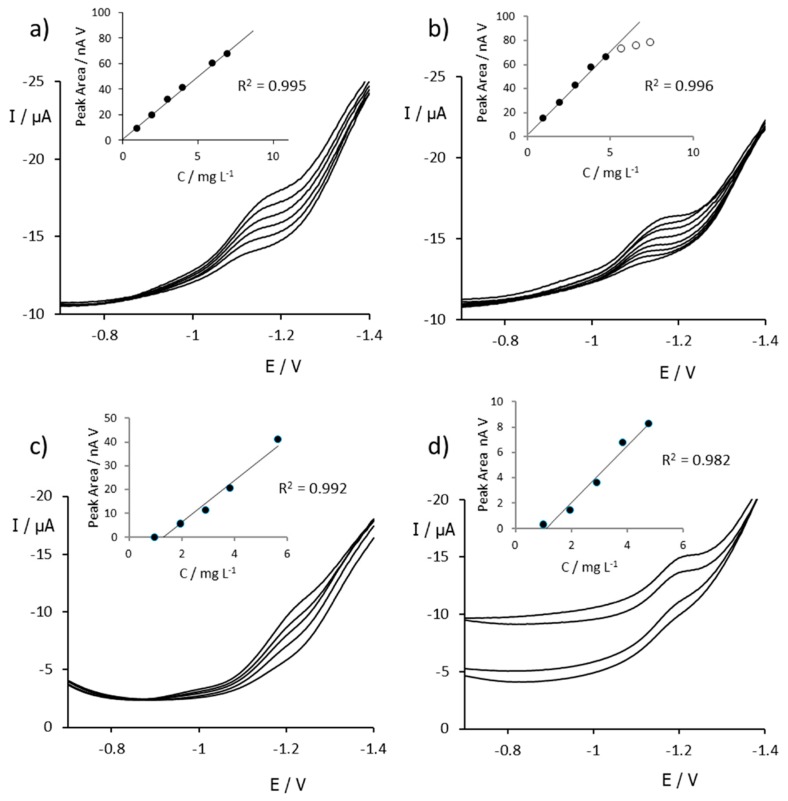
DPV voltammograms measured at pH 2.2 using an SPCNFE in BZT (**a**) and Me-BZT (**b**) solutions and using an SPCNTE in BZT (**c**) and Me-BZT (**d**) solutions. The insets show the corresponding calibration plots estimated using the peak areas and the R^2^ values obtained.

To obtain the calibration curves, DPV voltammograms were recorded at pH 2.2 using both SPCNFE and SPCNTE electrodes in standard solutions of BZT and Me-BZT at concentrations in the ranges of 1–8 mg L^−1^ for BZT and 1–6 mg L^−1^ for Me-BZT (since in the first experiments, Me-BZT showed electrode saturation at lower concentrations than BZT). These DPV voltammograms and the corresponding calibration plots are depicted in Figure 5, and Table 1 shows the analytical parameters computed from them. For LODs and LOQs, the values shown are the average values from several calibration plots. As an example of how they are computed, let us consider the calibration line shown in Figure 5a. It is described by the equation y = 9.80 x + 0.49 with R^2^ = 0.995 and a standard deviation for the intercept of 1.45. The LOD and LOQ are then computed according to LOD = 3 · 1.45/9.80 = 0.44 and LOQ = 10 · 1.45/9.80 = 1.48.

From Figure 5, it is clear that, as suggested by preliminary CV measurements, the signals of BZT and Me-BZT appear at practically the same potential when measured with the same electrode, either with the SPCNFE or with the SPCNTE. This means that in real samples it would be not possible to discriminate both benzotriazole isomers. In contrast, the signals are recorded at more negative potentials when the SPCNTE is used (ca. 50 mV more negative than in the case of the SPCNFE). Moreover, the baseline and signals of both BZTs appear better defined in SPCNFE measurements. Besides that, the linearity of the calibration plots in the insets is much clearer when using the SPCNFE (Figure 5a,b) as compared to the SPCNTE (Figure 5c,d), which generates negative intercepts. 

As shown in Table 1, the SPCNFE provides higher sensitivity and repeatability, lower LODs and LOQs, and wider linearity ranges for both analytes as compared to the SPCNTE. According to these results, the SPCNFE was finally selected for the further determination of BZTs in water samples. 

As an additional analytical parameter, the reproducibility of SPCNFE measurements was evaluated from the RSD of the slope of three calibration lines for BZT obtained on different days, giving a value of 4.8%. Comparing the obtained results in this study with those reported by the authors of [30,31], the LODs of the proposed methodology are slightly higher. However, this could be compensated by the fact that commercial screen-printed units are simpler and faster to use than the more tedious home-made modification of glassy carbon electrodes and are more environmentally friendly than mercury electrodes. In addition, to the best of our knowledge, these results constitute the first data on BZT determination in water using commercial SPEs. 

The proposed method, despite having LOQs in the low mg L^−1^ range, allows the on-site analysis of quite polluted samples, such as those reported for BZT by Cancilla et al. [25] near an airport (126 mg L^−1^) and by Lowenberg et al. [26], (6 mg L^−1^) in wastewater. Moreover, this method can be used as a useful tool in the identification of toxic concentrations of BZTs in waters because of the EC50 values for several aquatic organisms, i.e., *Vibrio fischeri*, *Daphnia magna*, *Daphnia galeata*, *Ceriodaphnia dubia*, and *Pimephales promelea* are in the range 8.7–107 mg L^−1^ [5].

### 3.3. Determination of Benzotriazole in a Spiked Water Sample

An extensive assessment on the applicability of the proposed methodology to different types of polluted water samples was outside the scope of the present work. Nevertheless, a preliminary test was made with real tap water samples spiked with BZT. Thus, we analyzed in triplicate water samples from the local distribution network, operated by Agbar Company (Barcelona; http://www.agbar.es/eng/home.asp) mostly from the water of the Llobregat River and collected from a conventional tap in the laboratory. The water samples were spiked with the corresponding aliquots of the BZT standard solution to 3 mg L^−1^ and were analyzed by DPV with SPCNFE using the standard addition method for quantification. The same SPCNFE sensor was used for the measurement of the original spiked sample and the sample with the successive additions of the BZT standard.

**Figure 6:**
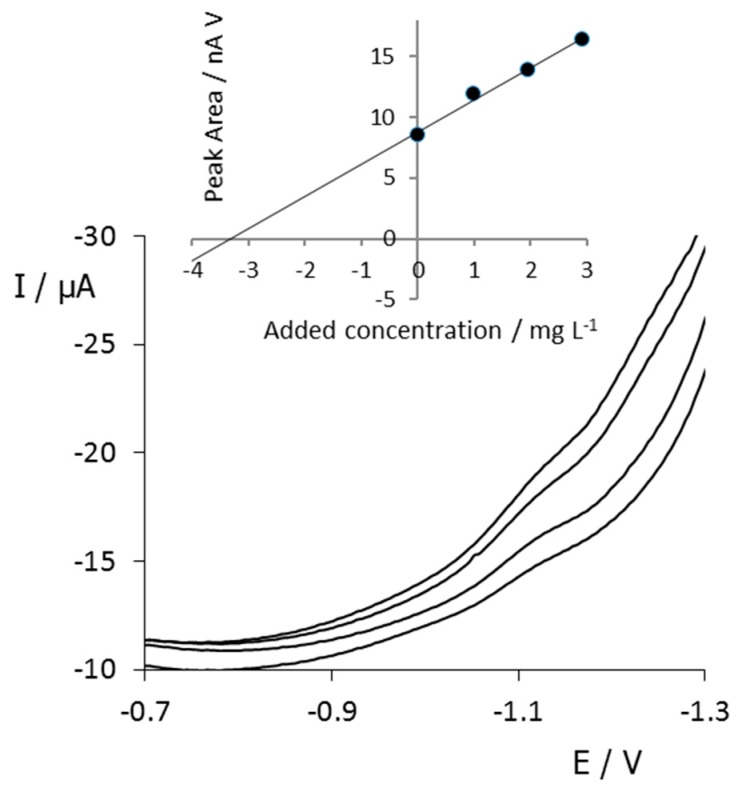
DPV voltammograms measured at pH 2.2 with an SPCNFE of a spiked tap water sample at 3 mg L^−1^ BZT and after three standard additions of BZT. The inset shows the calibration plot estimated based on the peak areas.

Figure 6 shows that the signal of BZT reduction was the only one registered within the working potential range, suggesting that no interfering substances from the matrix were present. The obtained results (see Table 2) suggest that BZTs could be successfully analyzed in environmental water samples at the very low mg L^−1^ range using electroanalytical techniques coupled to a preliminary concentration step. Consequently, DPV measurements using SPCNFEs could become a cheap, simple, faste, and portable complementary strategy to sophisticated and costly techniques such as HPLC-MS/MS.

## 4. Conclusions

This work suggests that DPV using commercial SPEs is a fast, simple, low-cost, reliable, and environmentally friendly methodology complementary to the expensive, high waste, and non-portable HPLC-MS/MS approach for the screening and on-site monitoring of BZTs in waters and wastewaters. Among the screen-printed electrodes tested, SPCNFEs exhibited the best analytical performance.

Although confirmation is required with other related molecules, the great similarity of the voltammetric signals of BZT and Me-BZT suggests that the proposed methodology cannot discriminate among different BZTs. It can, however, be successfully applied to evaluate the total BZTs content in a sample, or as an indicator of high BZTs contamination, leaving the further task of quantifying every single substance to HPLC-MS/MS. 

As for the detection and quantification limits, the values achieved in this work using SPCNFE are not far above those reported in previous studies using modified glassy carbon and mercury electrodes, with the advantage of being simpler, faster, cheaper, and much more environmentally friendly. However, these values are in the low mg L^−1^ range, which, generally, are far from the concentrations expected in environmental samples. Nevertheless, natural waters from highly human-impacted ecosystems (e.g., close to airports or in polar areas, where anti-freeze and deicing fluids are necessary) and wastewaters can accumulate high concentrations of BZTs. Besides, the presented DPV method could be applicable to trace or ultra-trace analysis with previous pre-concentration of the water samples by chemical and/or electrochemical means. 

To conclude, the present work claims the applicability of commercial screen-printed devices for the voltammetric sensing of benzotriazoles, thus encouraging research on electrode modifications with selective reagents to achieve detection limits below the usual BZTs concentrations found in environmental samples.

## Figures and Tables

**Table 1:** Performance of the DPV determination of BZT and Me-BZT by using an SPCNFE and SPCNTE computed from the calibration plots in Figure 5. The standard deviations of sensitivity are denoted in parenthesis. The results are compared with these in refs. [30,31]. LOD: limit of detection; LOQ: limit of quantification; RSD: relative standard deviation.

Electrode /Technique	Analyte	Sensitivity (nA V mg^−1^ L)	LOD (mg L^−1^)	LOQ (mg L^−1^)	Linear Range (mg L^−1^)	Repeatability (% RSD)	Reference
SPCNFE/DPV	BZT	9.8 (0.5)	0.4	1.2	1.2–8.0	4.3	This work
	Me-BZT	14.6 (0.7)	0.4	1.3	1.3–5.0	8.0	This work
SPCNTE/DPV	BZT	8.7 (0.2)	0.3	0.9	0.9–6.0	6.2	This work
	Me-BZT	11.2 (0.7)	0.9	3.0	3.0–5.0	13.9	This work
GCE+CNT +Nafion/SWV	BZT	-	0.09	-	0.4–19.0	-	[30]
SMDE/DPP	Me-BZT	-	0.05	-	0.4–30.0		[31]

**Table 2:** Comparison among the theoretical (spiked) concentration of BZT in a tap water sample and that determined with the proposed methodology using SPCNFE. Analyses were performed in triplicate, *n* = 3.

R^2^	Concentration Spiked (mg L^−1^)	Concentration Found (mg L^−1^)	Standard Deviation	Recovery (%)	Relative Error (%)
0.990	3.0	3.3	0.1	110	10
